# Classical sheep scrapie in Great Britain: spatial analysis and identification of environmental and farm-related risk factors

**DOI:** 10.1186/1746-6148-5-33

**Published:** 2009-09-08

**Authors:** Kim B Stevens, Victor J Del Río Vilas, Javier Guitián

**Affiliations:** 1 Veterinary Epidemiology and Public Health Group, Department of Veterinary Clinical Sciences, Royal Veterinary College, Hawkshead Lane, North Mymms, Hatfield, Hertfordshire, AL9 7TA, UK; 2 Centre for Epidemiology and Risk Analysis, Veterinary Laboratory Agency, Weybridge, New Haw, Addlestone, Surrey, KT15 3NB, UK; 3 Department for Environment, Food and Rural Affairs (Defra), Nobel House, 17 Smith Square, London, SW1P 3JR, UK

## Abstract

**Background:**

Previous studies suggest that the spatial distribution of classical sheep scrapie in Great Britain is uneven and that certain flock characteristics may be associated with occurrence of the disease. However, the existence of areas of high and low disease-risk may also result from differences in the spatial distribution of environmental characteristics. In this study we explored the spatial pattern of classical scrapie in Great Britain between 2002 and 2005 and investigated the association between disease occurrence and various environmental and farm-related risk factors.

**Results:**

*Exploratory spatial analysis*: South Wales was found to have a higher density of scrapie-positive farms than the rest of Great Britain. In addition, a small cluster of high-risk farms was identified in the center of this region in which clustering of scrapie-positive farms occurred up to a distance of approximately 40 km.

*Spatial modelling*: A mixed-effects regression model identified flock-size and soil drainage to be significantly associated with the occurrence of scrapie in England and Wales (area under the curve (AUC) 0.71 ± 0.01, 95% CI 0.68 - 0.74). The predictive risk map based on the estimated association between these factors and disease occurrence showed most of Wales to be at risk of being confirmed positive for scrapie with areas of highest risk in central and south Wales. In England, areas with the highest risk occurred mainly in the north and the midlands.

**Conclusion:**

The observed distribution of scrapie in Great Britain exhibited a definite spatial pattern with south Wales identified as an area of high occurrence. In addition both flock (flock size) and environmental variables (soil drainage) were found to be significantly associated with the occurrence of the disease. However, the model's AUC indicated unexplained variation remaining in the model and the source of this variation may lie in farm-level characteristics rather than spatially-varying ones such as environmental factors.

## Background

Classical scrapie, a transmissible spongiform encephalopathy (TSE) of small ruminants, has been endemic in Great Britain for at least 250 years [1]. As the occurrence of scrapie was known to be associated with certain family lines of sheep the disease was originally believed to be genetic and non-infectious [2]. However, classical scrapie is now known to be an infectious disease and it is accepted that for an animal to develop scrapie it has to have both a susceptible genotype and be exposed to the agent. Although there is evidence in support of scrapie being associated with the conversion of the host-encoded prion protein PrP^C ^to a misfolded, partially proteinase-K resistant isoform called PrP^SC^, the precise nature of the agent is still being investigated.

Classical scrapie was made a notifiable disease in January 1993 as required by European Union legislation [3] and since 1998 data for all statutorily reported clinical cases of scrapie in Great Britain must be recorded in the Scrapie Notifications Database (SND). The National Scrapie Plan (NSP) was introduced in 2001 in an attempt to eradicate the disease from Great Britain by increasing the prevalence of scrapie-resistant genotypes in the national flock. Since 2002 a new prion disease of sheep has been reported in the UK; atypical scrapie. This disease is distinct from classical scrapie as not only do the two diseases have different spatial patterns [4] but atypical scrapie occurs in sheep known to be genetically resistant to classical scrapie [5].

Some studies have suggested that certain flock characteristics may be associated with the occurrence of classical scrapie. In Great Britain, an analysis of data collected via a postal questionnaire found geographical region, flock-size and flock type to be significant risk factors for the occurrence of classical scrapie [6]. Large flocks and those with purebred sheep were at greater risk of experiencing the disease than small flocks or those with crossbred sheep. The same flock characteristics (size, and whether crossbred or purebred) and broadly-defined geographical regions were identified as risk factors by the follow-up survey of 2002 [7,8].

The geographical variation in risk of disease is supported by a more recent study which, working at a higher level of resolution (using the point location of the farm), identified certain regions in Britain as having a higher or lower than average risk of disease [9]. This last study used data from the period prior to the 2001 UK foot and mouth disease (FMD) epidemic, which had a large impact on the structure of the livestock sector in the UK. Since then the number of farms reporting to the SND has decreased considerably [10].

Studies conducted in Norway [11], Ireland [12], France [13] and Iceland [14] identified a series of factors associated with an increased risk of occurrence of classical scrapie: purchase of female sheep from scrapie-infected flocks, sharing of rams and sharing of pastures between flocks [11], large breeding-flocks, purchase of replacement sheep from markets, the spreading of sheep compost on land [12], using concentrates and milk replacements [13] and the ratio of iron-to-manganese in forage grown on scrapie-affected farms [14]. Binding of the scrapie causal agent to some basic elements, such as copper [15], led to investigations of the association between the occurrence of the disease and the presence of soil trace elements. Although a British study found no such relationship [16], Gudmundsdottir *et al. *[14] found that in Iceland both the concentration of iron, and the ratio of iron-to-manganese in forage was greater in scrapie-affected than non-affected farms. Imrie *et al. *[17] has shown the spatial distributions of bovine spongiform encephalopathy (BSE) and classical scrapie to be similar in Great Britain and suggested this might be associated with increased soil pH and total organic carbon, and decreasing soil iodine concentrations.

The available evidence therefore suggests that certain flock characteristics may be associated with the risk of classical scrapie and that the geographical distribution of the disease is uneven. The existence of areas of high and low risk of disease may be the result of differences in the spatial distribution of environmental influences or regional variation in farms' characteristics. In addition farmers' reporting behaviour may influence estimates of frequency of disease, and thereby the detection of areas of high or low risk [18].

The aim of this study was twofold: firstly, to visualize and explore the spatial pattern of classical scrapie in Great Britain between 2002 and 2005 and secondly, to investigate the association between selected environmental and farm-related risk factors and the occurrence of the disease. It is expected that a better understanding of the spatial distribution of the disease and of the factors underlying the observed spatial patterns may provide useful information for the targeting of surveillance and control efforts.

## Methods

### Study area, study population and study design

The study area comprised Great Britain. All sheep farms included in the 2004 Agricultural Census and identified by a unique CPH (County, Parish, Holding) number were included in the analysis as the baseline population (n = 78 157). 1882 (2%) farms were excluded from the baseline population as they lacked a valid CPH. The 78 157 farms included in the study were subdivided into those reported to the SND between 1^st ^January 2002 and 31^st ^December 2005 (n = 666) and those not reported to the SND (n = 77 491). The 666 farms that had been reported to the SND were classified as being either scrapie-positive when at least one submission tested positive (n = 411) or scrapie negative otherwise (n = 255).

For the purpose of this study we carried out a retrospective comparison of case and non-case farms. Cases were defined as all farms reported to the SND between 1^st ^January 2002 and 31^st ^December 2005 and were and where at least one submission tested positive for scrapie. Non-case farms were defined as farms that did not report any instances of suspected scrapie to the SND between 1^st ^January 2002 and 31^st ^December 2005. For the purpose of analysis a sample of non-case farms was randomly selected from all non-case farms. For all analyses in this study the ratio of case to non-case farms was 1:4.

None of the randomly-selected sample of non-case farms had been detected positive by the fallen stock surveillance scheme between 2002 and 2005. However, as a result of traceability issues it was not possible to check whether farms detected positive by the abattoir surveillance scheme were amongst the randomly-selected sample of non-case farms.

### Data sources

#### Farm location

Farms were assigned Cartesian coordinates based on their CPH in the 2004 UK Agricultural Census. If no CPH was available farms were assigned the coordinates of their postcode (if available). Coordinates derived from CPHs or postcodes were checked to ensure they fell within the correct parish as indicated by the farm's CPH number. If neither CPH nor postcode were available, farms were assigned the coordinates of their parish centroid. A total of 65 632 farms (84.1%) were assigned coordinates based on either CPH or postcode, and 12 417 (15.9%) were assigned the coordinates of the parish centroid.

#### Farm-level data

For all farms included in the study the flock-size (number of adult sheep on the farm) and farm-area (ha) were extracted from the 2004 UK Agricultural Census. Farm altitude (meters above sea level) was extracted from a digital terrain map with a 50 m grid derived from Ordnance Survey landform data. Stocking density was calculated as the number of adult sheep divided by the area of the farm.

#### Disease data

The following data were extracted from the SND for each farm that had reported at least one suspect case to the SND: date of first report and date of first confirmed positive case. No information was available on the genotype of the flocks.

#### Environmental data

Environmental variables were selected for inclusion in the model based on data availability and biological plausibility of a relationship with scrapie. For all farms in the study population the following data were extracted from their respective geographic data sources: mean annual rainfall (mm) for 2002 to 2005, mean annual temperature (°C) for 2002 to 2005, soil drainage, soil texture (i.e. clay, sand, loam or peat), soil fertility, and land cover characteristics. Mean annual rainfall and temperature data for 2002 to 2005 were obtained from the United Kingdom Met Office 5 km × 5 km gridded data sets . The raster maps showing mean monthly temperature (or rainfall) from January 2002 to December 2005 were summed and divided by 48 resulting in a map of the mean annual temperature (or rainfall) for Great Britain for the period 2002 to 2005. The location of all farms in the study population was superimposed on the temperature and rainfall maps and the mean annual temperature and rainfall for 2002 to 2005 extracted for each farm using the intersect-point tool available as part of Hawth's Analysis Tools for ArcGIS . Land cover data were obtained from the 1 × 1 km resolution raster Land Cover Map 2000  in a similar manner. Soil drainage, texture and fertility data were extracted from the 1:250 000 NATMAP SoilScapes map for England and Wales  by superimposing the location of all farms in the study population on the SoilScapes map and using the point-in-polygon tool available in ArcGIS 9.2 to extract the soil attributes for each point. For the purpose of analysis, the 12 original soil fertility categories were collapsed into five categories (high, moderate, low, very low, lime-rich) and the 6 original soil drainage categories were collapsed into three (freely draining, impeded drainage, naturally wet).

### Statistical and spatial analysis

#### Summary statistics and temporal patterns

Descriptive statistics were obtained for all continuous variables under consideration (flock-size, farm-area, farm altitude, stocking density, mean annual temperature, mean annual rainfall) using SPSS 16.0 for Windows (SPSS Inc., Chicago, Illinois, USA). The Mann-Whitney U test of association was used to identify significant differences between case and non-case farms for each variable. To visualize the temporal pattern of reporting to the SND throughout the study period, the time to reporting was graphed and compared between flocks reporting to the SND in England, Scotland and Wales using the Breslow test of association.

#### Kernel-smoothed maps of farm density

All maps were produced in ArcGIS 9.2 (ESRI, Redlands, CA, USA). Kernel-smoothed maps showing the density of all sheep farms in Great Britain (the baseline population), and of case farms were produced by applying kernel estimation to the location distributions. Optimum bandwidths for England, Scotland and Wales were estimated individually by means of the quartic approximation of a true Gaussian kernel function using least-squares cross-validation [19] and the normal optimal smoothing method [20] implemented using the SM and MASS packages in R . The bandwidth values obtained by normal optimal smoothing were consistently higher than those obtained by least-squares cross-validation. A conservative approach was adopted as higher bandwidths that would tend to over-smooth and reduce the chances of over-interpretation were preferred, although this reduced our ability to detect small spatial variations in risk. As the estimated optimum bandwidths for Welsh case farms differed considerably to those of farms in England and Scotland, Wales was considered separately from England and Scotland when generating the kernel-smoothed maps.

Bandwidths of 20 km were used to create the kernel-smoothed maps of both case and non-case farms in Wales, and bandwidths of 60 and 45 km for case and non-case farms respectively in both England and Scotland. An output cell size of 2.5 km^2 ^was used.

#### Kernel density ratio maps

In order to adjust for the underlying baseline population, maps showing the distribution of the odds ratio of farms confirmed positive for scrapie were obtained. The kernel-smoothed surfaces for farms confirmed positive for scrapie (per square km) were divided by the kernel-smoothed density surface of the non-case farms (per square km). Given the overall ratio of cases to non-cases (1:4) a result of 0.25 for the division of both surfaces was interpreted as OR = 1.

#### Clustering and cluster detection

The spatial scan statistic [21] was used to identify significant clusters of farms with a high or low risk of being confirmed positive for scrapie, and was implemented in SaTScan v7.0.3 using a Bernoulli probability model, a circular scanning window set to contain a maximum of 50% of the population at risk, and Monte-Carlo randomisation with 999 permutations.

Within the most likely cluster (as identified by the spatial scan statistic), Ripley's *K*-function test [22] was used to identify the scale at which clustering of case farms occurred, in relation to non-case farms. As Ripley's *K*-function test takes into account the distribution of the baseline population any significant clustering identified by the test indicates significantly more cases relative to non-cases in the area of interest. Monte-Carlo randomisation with 99 simulations was used to randomly permute the locations of case and non-case farms, and the upper and lower bounds of these permutations were plotted together with the observed difference function. The analysis was implemented using the SPLANCS package [23] in R 2.7.1 .

### Spatial modelling

#### Identification of risk factors

The spatial modelling focused on England and Wales as, at the time of the study, no digitized soil map comparable to the SoilsScapes map for England and Wales was available for Scotland. The dataset for the spatial modelling therefore contained 224 scrapie-positive farms (England n = 119; Wales n = 105). All continuous variables were converted to categorical ones based on quartiles. To identify risk factors for farms being confirmed positive for scrapie in England and Wales a mixed-effects logistic regression model was fitted to the data using the GLIMMIX procedure available in SAS 9.2 (SAS Institute Inc, Cary, NC, USA). Initially, the following potential predictor variables were individually assessed in a univariable binomial logistic regression model: flock-size, farm area, stocking density, whether the production system was sheep only or cattle and sheep, altitude, mean annual rainfall, mean annual temperature, soil texture, soil drainage, soil fertility, land cover, region and county. All variables that achieved an alpha level of 0.2 in the univariable logistic regression model (apart from region and county) were initially included in the multivariable mixed-effects model. As the variable region was more strongly associated with the outcome than the variable county (p = 0.05 versus p = 0.08), region was included in the model as a random effect to account for large-scale (first-order) spatial variation in the data. Proc GLIMMIX uses quasi-likelihood methods for estimation [24] which results in a log-pseudo likelihood and therefore standard likelihood ratio tests and information criteria are not considered valid indicators of model fit. Variable selection for the final multivariate model was therefore based on a manual backward selection procedure (alpha level of 0.05) in which each variable was removed in turn starting with the variable with the highest p-value. The effect of removing each variable was considered by examining the change to the estimates, standard errors and p-values of the remaining variables. All first-order interaction terms of the variables remaining in the final model were similarly assessed for significance. The stability of the final model was assessed by returning eliminated variables to the model individually and examining the change to the estimates, standard errors and p-values of the final model variables.

In order to determine whether there was any small-scale (second-order) spatial variation in the data the final multivariate model with region as a random effect was then re-run with an exponential spatial covariance structure incorporated. For both models, semivariograms of the model residuals with a simulation envelope based on 99 Monte Carlo permutations were produced using R and the geoR and geoRglm packages [25]. These were visually appraised to determine the existence of significant spatial dependency and the distance up to which case-farms were correlated. As inclusion of the exponential spatial covariance structure had no effect on the model estimates, standard errors and p-values, or the semivariogram of the residuals, the model without the exponential spatial covariance structure was retained as the final model.

For the final model, directional semivariograms at angles of 0, 45, 90 and 135° (with a tolerance of 22.5°) were plotted to determine whether the spatial distribution of the model residuals varied with direction. The predictive ability of the model was assessed by producing a receiver operating characteristic (ROC) curve comparing the actual and predicted status of farms, and by calculating the area under the curve, its associated standard error and 95% confidence interval.

#### Risk mapping

The final model resulting from the mixed-effects logistic regression was fitted to all sheep farms in England and Wales thereby obtaining a risk value for all point locations. As the directional semivariogram showed the spatial distribution of the model residuals did not vary with direction, ordinary kriging was used to convert the point risk-values into a continuous risk surface, with an associated standard error map.

## Results

### Descriptive statistics

Of the 411 farms confirmed positive for scrapie during the study period 245 (60%) farms reported to the SND for the first time while 166 (40%) farms had previously reported confirmed cases to the SND. 56.2% (n = 231) were in England, 35.8% (n = 147) in Wales, and 8.0% (n = 33) in Scotland. Summary statistics for the 411 case and 1644 randomly-selected non-case farms are presented in Table 1. In all instances scrapie-positive farms had significantly larger flocks and a larger farm area than scrapie-negative farms.

**Table 1:** Summary statistics and univariable comparisons using the Mann-Whitney U test, for the variables flock-size, farm-area, density, altitude, mean annual temperature and mean annual rainfall for farms confirmed scrapie-positive (n = 411) and a random sample of scrapie-negative farms (n = 1644) in Great Britain between 1^st ^January 2002 and December 2005.

**Variables**	**Scrapie positive** **(n = 411)**		**Scrapie negative** **(n = 1644)**		**P-value** **(Mann-Whitney U test)**
	
	**Median**	IQR*	**Median**	IQR*	
**Altitude (masl)**					
England	140	124	140	136	0.41
Scotland	86	128	137	151	0.006
Wales	282	139	241	199	0.002
**Flock size (no. sheep)**					
England	312	452	61	196	<0.001
Scotland	330	479	98	315	<0.001
Wales	861	764	193	456	<0.001
**Farm area (ha)**					
England	78.8	105.6	22.4	77.6	<0.001
Scotland	129	224.4	36.5	152.7	0.001
Wales	117.5	101.43	42.5	81.9	<0.001
**Density (sheep/ha)**					
England	4.2	5.5	2.9	4.8	<0.001
Scotland	2.7	3.7	2.6	4.7	0.637
Wales	7	3.7	5.2	4.6	<0.001
**Mean temperature 2002 - 2005 (°C)**					
England	10	1.32	10.1	1.06	0.306
Scotland	8.7	0.55	8.8	1.01	0.544
Wales	9.5	1.18	9.8	1.23	0.014
**Mean rainfall 2002 - 2005 (mm)**					
England	73.4	37.6	69.6	31.2	0.204
Scotland	82	19.59	98.7	39.4	<0.001
Wales	112.1	46.72	103.9	35	0.006

* IQR = interquartile range

**Figure 1:**
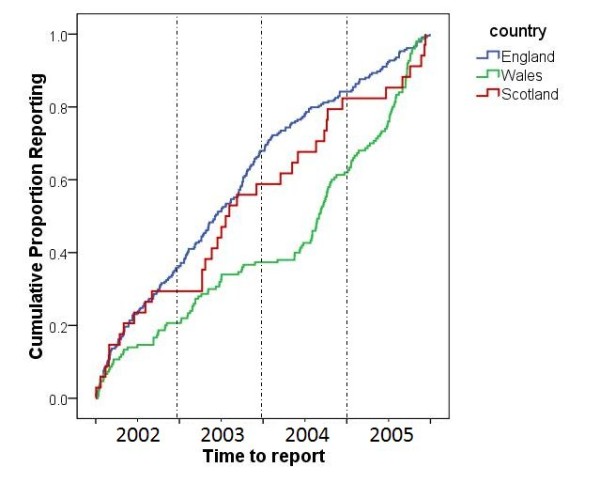
**Time-to-reporting for 443 farms in England, Scotland and Wales, reporting to the Scrapie Notifications Database (SND) for the first time between January 2002 and December 2005**.

The temporal pattern of farms reporting to the SND (irrespective of whether or not they were confirmed positive for scrapie) differed significantly between England, Scotland and Wales (Breslow test: *P *< 0.001). Although reporting patterns were similar for England and Scotland throughout the study period, Wales had a relatively low reporting rate between 2002 and mid-2004 with only 20% of reported cases occurring during this period, but from 2004 onwards the Welsh reporting rate accelerated (Figure 1).

### Risk of farms being confirmed positive for scrapie

**Figure 2:**
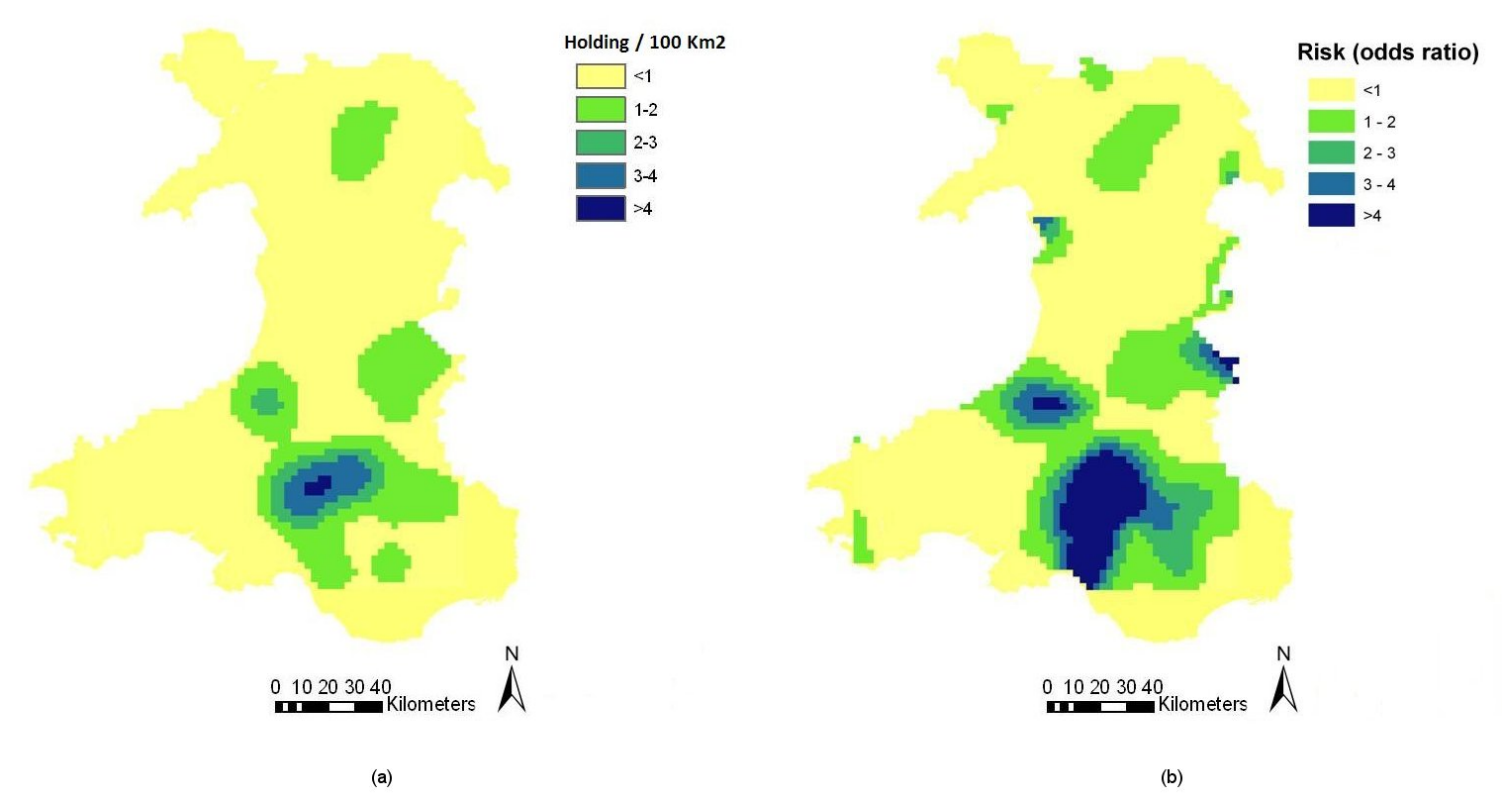
**(a) Kernel-smoothed map showing density of sheep farms confirmed positive for scrapie (20 km bandwidth) and (b) kernel-density ratio surface displaying the odds of a farm in Wales being scrapie-positive**.

**Figure 3:**
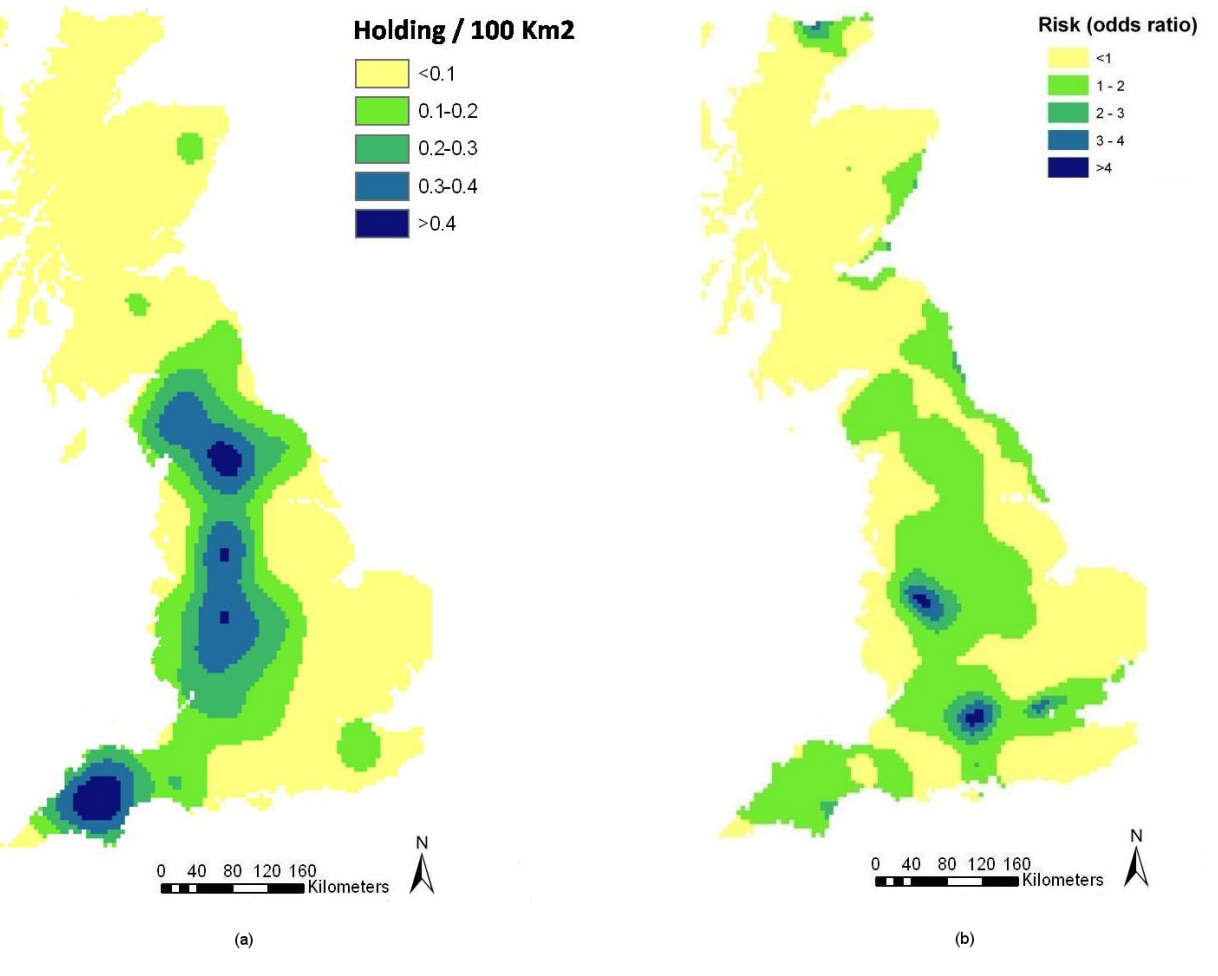
**(a) Kernel-smoothed map showing density of sheep farms confirmed positive for scrapie (60 km bandwidth) and (b) kernel-density ratio surface displaying the odds of a farm in England-Scotland being scrapie-positive**.

Kernel smoothed density surfaces for Wales and England-Scotland are presented in Figures 2a and 3a respectively while kernel density ratio surfaces displaying the distribution of the odds ratio of farms being confirmed positive for scrapie in Wales and England-Scotland, are presented in Figures 2b and 3b respectively. These maps identified south Wales as a high-risk region (Figure 2b). In contrast, the risk of farms being confirmed positive for scrapie in England-Scotland was lower (Figure 3b).

### Cluster detection

**Figure 4:**
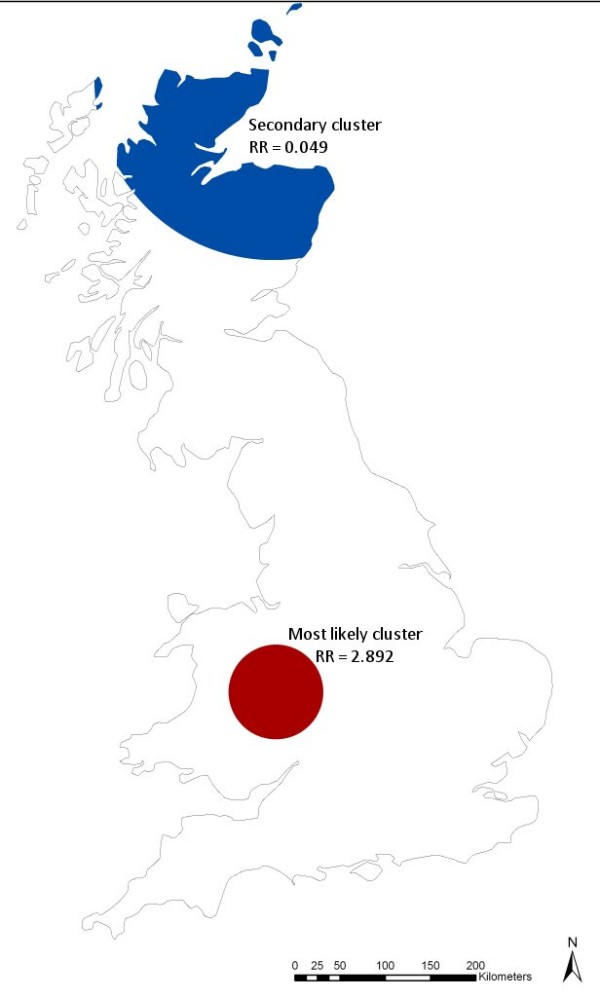
**Position of significant (p < 0.05) clusters of farms in Great Britain with a high or low relative risk of being confirmed positive for scrapie (RR = relative risk)**.

**Figure 5:**
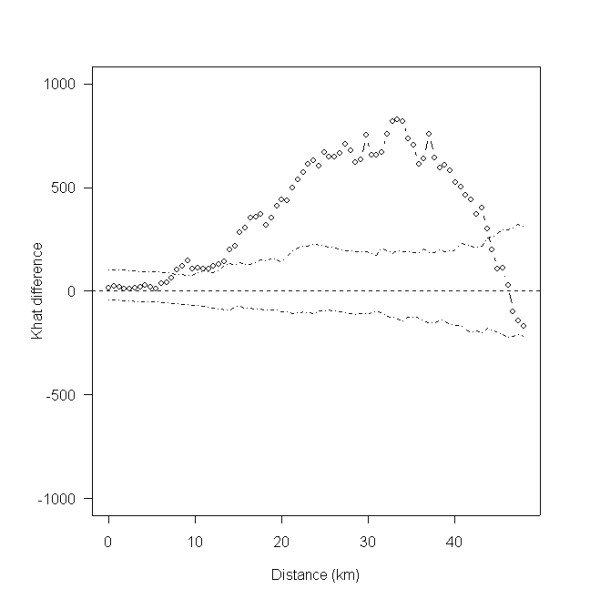
**Difference between *K*-functions for case (scrapie positive) and non-case farms within the most likely cluster identified by the spatial scan statistic (as illustrated in Figure 4)**.

The spatial scan statistic identified one significant cluster (relative risk = 2.892; p = 0.001) of farms with a high risk of being confirmed positive for scrapie in central Wales and one significant cluster of farms with a low risk of being confirmed positive for scrapie in northern Scotland (relative risk = 0.049; p = 0.001). Their locations are shown in Figure 4 and their characteristics are presented in Table 2. The results of Ripley's K-function test established that, within the most likely disease cluster there was significant small- to medium-scale clustering of scrapie-positive farms (between 8 and 42 km; Figure 5).

**Table 2:** Characteristics of the significant (p < 0.05) high- and low-risk clusters in Great Britain, as identified by the spatial scan statistic based on a Bernoulli probability model and using a circular scanning window to include 50% of the population at risk (as illustrated in Figure 4)

**Cluster**	**Observed Cases**	**Expected Cases**	**Total holdings in cluster**	**Relative risk**	***P*-value**
Most likely cluster	104	43	216	2.892	0.001
Secondary cluster	2	37	186	0.049	0.001

### Identification of risk factors for farms confirmed positive for scrapie

**Figure 6:**
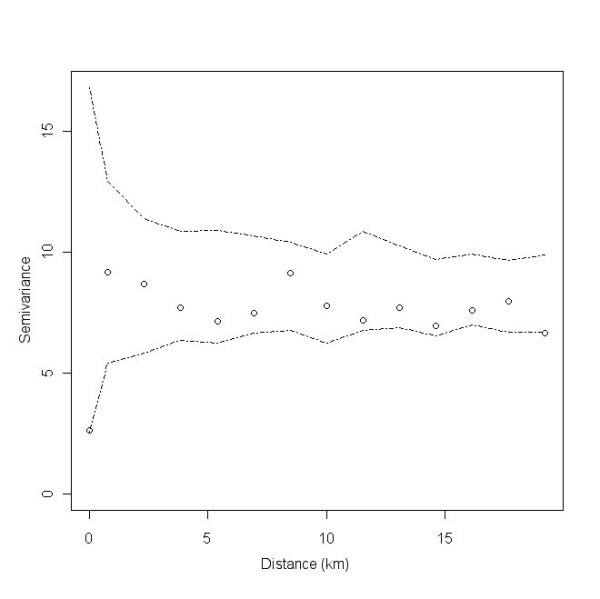
**Empirical semivariogram with Monte Carlo simulation envelope (dotted lines) of the residuals of the mixed-effects logistic regression presented in Table 3**.

The final multivariable mixed-effects logistic regression model identified flock-size and soil drainage to be significantly associated with occurrence of scrapie (Table 3). Compared to large flocks (>335 sheep), smaller flocks were all less likely to be confirmed-positive for scrapie, although this relationship was not linear. In addition, farms on naturally wet soils were almost twice as likely to be positive for scrapie than those on freely draining soils (OR 1.80, 95% CI 1.02 - 3.17) yet those on soils with impeded drainage were slightly less likely to be positive for the disease (Table 3). The area under the ROC curve (AUC) used to validate the model's predictive ability and the associated standard error was 0.71 ± 0.01 (95% CI 0.68 - 0.74). An empirical semivariogram of the model residuals is presented in Figure 6; all points are within the simulation envelope indicating no significant spatial dependency among the model residuals.

**Table 3:** Variables associated with farms in England and Wales being confirmed positive for scrapie

**Variable**	**Value**	**OR**	**95% CL**	***P*-value**
Flock-size	1 - 19	0.29	0.21 - 0.40	< 0.0001
	20 - 89	0.13	0.09 - 0.19	
	90 - 335	0.35	0.26 - 0.47	
	> 335	reference		
Soil drainage	Freely draining	reference		0.045
	Impeded drainage	0.89	0.64 - 1.24	
	Naturally wet	1.80	1.02 - 3.17	
Soil texture	Sand	reference		0.119
	Loam	0.57	0.28 - 1.14	
	Peat	0.92	0.41 - 2.10	
	Clay	1.08	0.30 - 3.86	
Soil fertility	High	reference		0.173
	Moderate	1.38	0.59 - 3.22	
	Low	1.14	0.53 - 2.47	
	Very low	0.52	0.16 - 1.71	
	Lime-rich	0.68	0.26 - 1.82	

### Risk map for occurrence of scrapie in England and Wales

**Figure 7:**
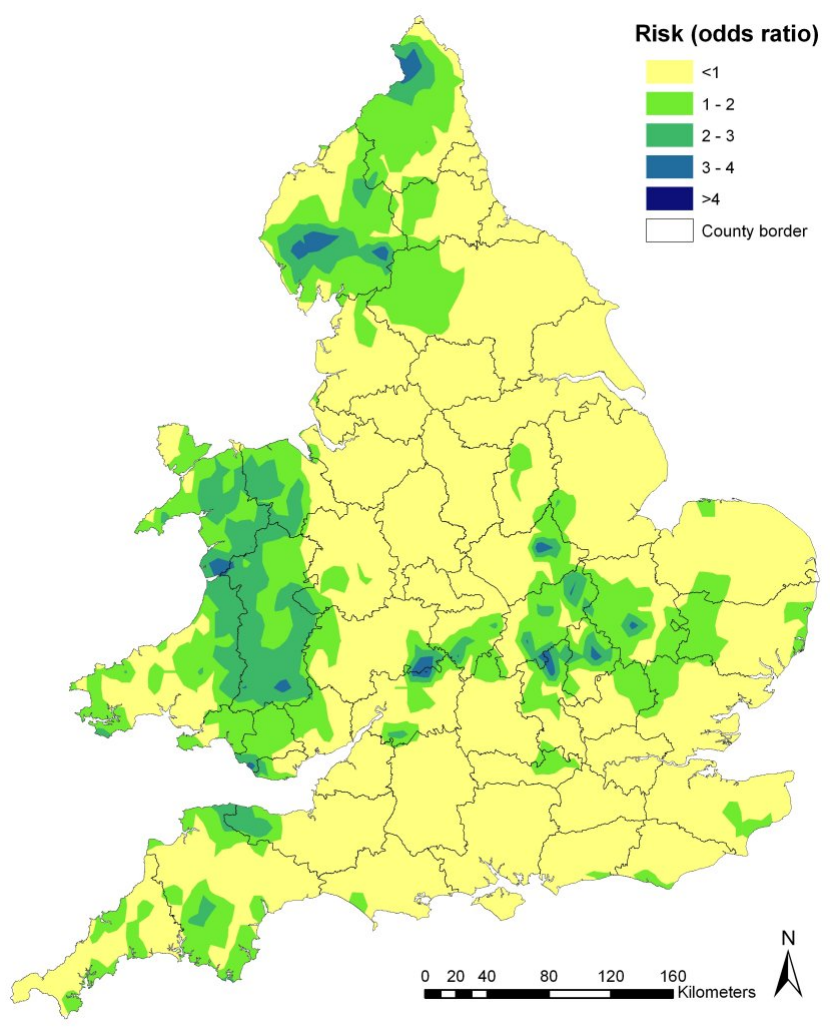
**Map showing the distribution of the predicted risk for the occurrence of scrapie in England and Wales, generated using the mixed-effects logistic regression model presented in Table 3**.

**Figure 8:**
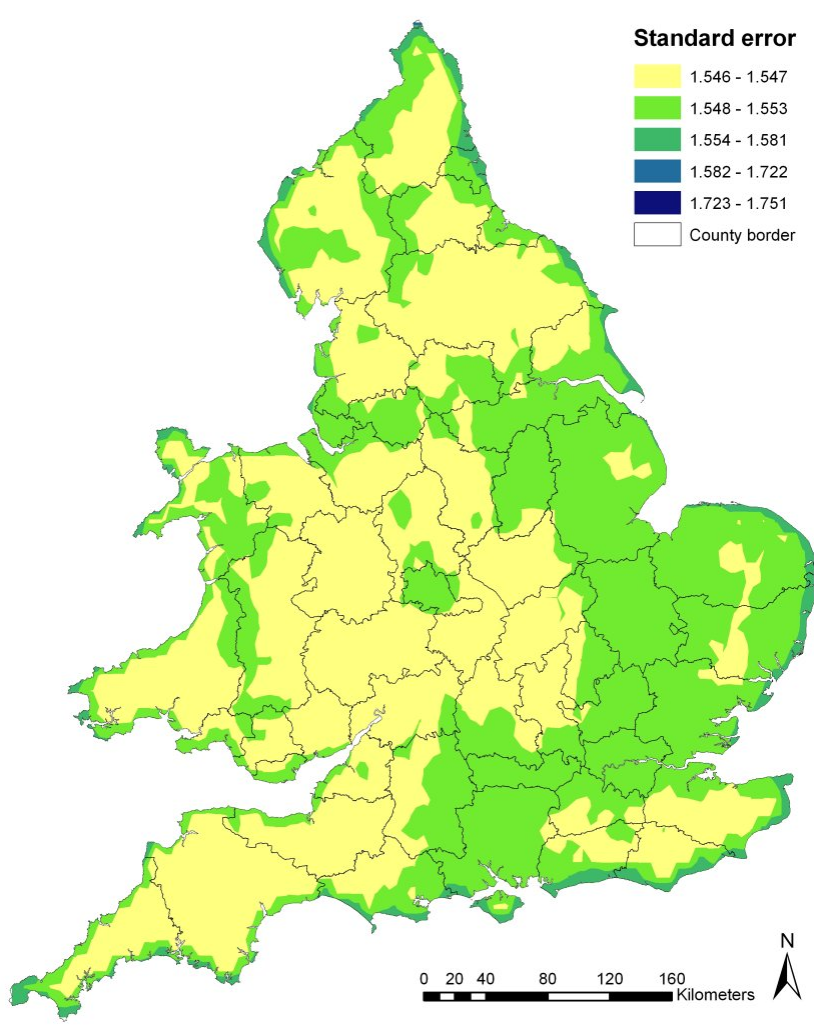
**Standard error map associated with the predicted risk of scrapie occurrence in England and Wales, as presented in Figure 7**.

Using the regression model presented in Table 3, a risk map was produced based on the estimated association between the identified risk factors and disease occurrence in England and Wales. This showed most of Wales to be at risk of being confirmed positive for scrapie with areas of highest risk in Powys, Gwynedd and Clywyd (Figure 7). In England, areas at risk of being confirmed positive for scrapie occurred mainly in the north (Cumbria, Northumberland and North Yorkshire) and in the Midlands (Leicestershire, Northamptonshire, Warwickshire, and Hereford and Worcester) and East Anglia (Cambridgeshire, Bedfordshire and Suffolk), with small pockets of high risk in the south-west (parts of Devon, Somerset and Cornwall; Figure 7). As illustrated in Figure 8 the standard errors associated with the risk values covered a small range (1.55 - 1.75) and were generally higher in the east than in the west of the study area, where the risk values were based on fewer point locations.

## Discussion

### Risk factors for scrapie

Unlike infectious diseases which display very marked spatial and temporal trends as they spread rapidly between locations, any spatial or temporal trend displayed by a disease such as scrapie, with a low between-flock transmission [26], is possibly due to the existence of common risk-factors at the farm-level. This study identified flock-size and soil drainage to be significantly associated with the occurrence of scrapie. Although we are not the first to identify large flocks to be a risk factor for the occurrence of scrapie [4,6,8,27] the interpretation of this result is difficult as the association between flock-size and the occurrence of scrapie may be direct or indirect. A larger number of sheep implies an increased number of susceptible animals, but on the other hand flock-size may be a proxy for a variety of management or biosecurity factors such as type of production system. Furthermore, in accordance with the work of del Rio Vilas *et al *[27], this study showed the relationship between occurrence of scrapie and flock-size to be non-linear.

The results of this study showed there to be an association between occurrence of scrapie and certain soil characteristics, in particular soil drainage. Other studies have also pointed to the existence of an association between soil geochemistry and scrapie although the findings have been inconsistent [16,17,28-30]. Imrie *et al *[17] suggested that the relationship may be an indirect one; the result of trace element deficiencies caused by decreased bioavailability of certain of these elements with increasing soil pH, total organic carbon and clay fraction. However, the results of Johnson *et al*'s [31] laboratory-based experiments provide another possible explanation for the association. Johnson *et al *[31]established that prion proteins adsorb tightly to clay particles but less readily to sand particles, suggesting that prion proteins could remain in the upper levels of clay soils and thus be readily ingested by sheep and other animals, while those in sandy soils are more likely to be leached away. Although the results of this, and other studies, suggest that soil may act as an environmental reservoir of the disease the exact mechanism remains to be determined.

Despite this and other studies pointing to the existence of an association between soil and scrapie this apparent relationship needs to be interpreted with caution bearing in mind certain limitations. Most importantly, we assigned soil attributes to the farm's georeference (either the farm's CPH, postcode or the parish centroid) which is a limitation of this study especially when using the parish centroid as the georeference or when the farm is composed of widely-spaced parcels of land. In addition, the apparent relationship between soil and scrapie may also be influenced by the time the animals spend grazing or exposed to the soil which may change between production types, breeds or seasons.

Epidemiological studies which attempt to identify risk factors associated with disease occurrence without accounting for the correlated nature of the data may obtain inaccurate results and wrongly conclude that an association exists between a potential risk factor and the outcome [32]. Spatial variation can occur at either, or both, the large- (first-order effects) or small-scale (second-order effects). Regional differences in the occurrence of scrapie have been observed in this and previous studies [6,7,9,27]. However, rather than model the actual differences in disease occurrence between regions we chose instead to include region as a random effect thereby accounting for large-scale variation in the spatial distribution of scrapie. The semivariogram of the model residuals showed there to be no unaccounted-for small-scale spatial variation in the remaining unexplained component of disease risk. This suggests that, after adjusting for the variation between regions and the effects of flock-size the source of the remaining unexplained variation in our model may lie in farm-level characteristics rather than in spatially-varying ones such as environmental factors. For example, as scrapie is known to be more prevalent among specific genotypes, it would be interesting to include this farm-level characteristic to determine how much of the variation in our model is explained by this variable.

### Spatial analysis versus risk mapping

This study shows that the distribution of cases of scrapie in Great Britain exhibits a definite spatial pattern. South and central Wales were identified as areas with a generally higher occurrence of the disease than the rest of Great Britain and a small cluster of high-risk farms was identified in this area. While the maps displayed in Figures 2b, 3b and 7 can all be seen as 'risk maps', Figures 2b and 3b show areas of high and low risk based on the actual spatial distribution of scrapie-positive farms while Figure 7 shows areas of predicted high and low risk based on the estimated association between the identified risk factors and the occurrence of disease. The fact that both the actual and predictive risk maps identify Wales to have the highest risk for scrapie suggests that the factors we identified as being associated with the occurrence of scrapie-positive farms are plausible.

However, in contrast to this study previous studies have found Wales to have a low-risk for scrapie [6,7,9,27]. This apparent contradiction may be largely due to the significantly different levels of reporting between England, Scotland and Wales during the study period. In England and Scotland almost half the cases were reported in the first half of the study period, yet during the same period only 20% of the Welsh cases were reported. Cases in Wales thus represent mainly the period 2004 to 2005. It is unlikely that such a sudden and dramatic increase in the level of Welsh-reporting is indicative of a correspondingly large increase in the occurrence of disease. The increased rate of reporting is more likely to be a reflection of a change in circumstances which encouraged a sudden increase in reporting from Welsh farmers. The launch of the Compulsory Scrapie Flocks Scheme (CSFS) in July (England and Scotland) and November (Wales) 2004 [33], coincided loosely with the increased rate of reporting in Wales. The compensation packages paid at the start of the CSFS might have influenced the reporting behaviour of farmers. If the increased rate of reporting from Welsh farmers in 2004 was the result of implementing the CSFS, it is unclear why a similar response was not observed in the reporting rates of farmers from England and Scotland.

### Informing surveillance

Spatial epidemiological studies such as this one having the potential to inform targeted surveillance systems as extraction mapping and cluster detection tests can be used to identify high-risk areas in which to focus surveillance efforts. On the basis of this study it would seem logical to focus surveillance efforts for scrapie in Wales, in particular in the high-risk areas and disease cluster we identified. However, it should be borne in mind that previous studies have found Wales to be a low-risk area for scrapie and the reason for this discrepancy needs to be explored in greater detail before the results of this study can be confidently used to inform scrapie surveillance in Great Britain.

### Limitations and biases

A major limitation of studies aimed at identifying associations between potential risk factors and disease lies in the difficulties and likely biases inherent in the identification of cases. This study is no exception. Inclusion of cases of scrapie in the SND database relies on cases being reported and therefore the results of this study have to be interpreted taking into consideration the source of the disease data and the associated, unmeasured reporting bias. For example, schemes and initiatives such as the NSP, Voluntary Scrapie Flocks Scheme (VSFS) and the CSFS may well have a spatially heterogenous impact on reporting rates which could influence estimates of disease frequency and the detection of areas of high or low risk. Previous studies that identified possible spatial heterogeneities in the frequency of the occurrence of scrapie in Great Britain have also been subject to reporting bias [6,7,9]. In fact, an American study concluded that over 80% of the variability in the incidence of scrapie in the United States was the result of reporting artefacts [18].

Three commonly used methods of georeferencing were used in this study; CPH, postcode and parish centroid. In principle, a method of georeferencing that assigns the centroid of a polygon as the point location of a farm may create problems (misclassification bias). However, for studies such as ours, performed at a low level of resolution (regional or national level) it has been shown that a random point in the parish is likely to be a sufficiently accurate method for the purpose of statistical analyses [34]. Another possible source of misclassification bias occurred when the soil texture and drainage categories were collapsed. However, as this misclassification was non-differential it would have reduced the strength of the association between soil drainage and fertility and the outcome [35].

Potential edge effects have not been formally addressed in the spatial analysis as it would have been computationally intensive and unlikely to have influenced the main patterns that were identified. However, the density maps should be interpreted with this in mind, especially for areas near the coastline.

## Conclusion

The results of this study show that the distribution of scrapie in Great Britain exhibits a definite spatial pattern with Wales, in particular south and central Wales, having a generally higher occurrence of the disease than England or Scotland. Flock-size and soil drainage were significantly associated with the occurrence of scrapie in England and Wales, and a risk map based on the estimated association between these factors and disease occurrence showed Wales and parts of England (the north and midlands) to have the highest predicted risk for scrapie. However, there is unexplained variation remaining in our model, the source of which may lie in farm-level characteristics rather than spatially-varying ones such as environmental characteristics. Future research might consider investigating this source of variation further, or conducting small-to medium-scale studies or performing similar analyses using data from the active surveillance programme in an attempt to confirm the results of the current study. However, the low numbers of farms detected though the active surveillance programme would make this comparison difficult at the current stage.

## Competing interests

The authors declare that they have no competing interests.

## Authors' contributions

KBS and FJG were responsible for the analysis of the data, interpretation of the results and the writing of the manuscript. All authors were responsible for the conception of the study. All authors read and approved the final manuscript.

## References

[B1] Stamp JT (1962). Scrapie: a transmissible disease of sheep. The Veterinary Record.

[B2] Parry HB (1984). Scrapie.

[B3] Anonymous (1991). Council Directive 91/68/EEC of 28 January 1991 on animal health conditions governing intra-community trade in ovine and caprine animals. Official Journal of the European Union.

[B4] Green DM, Del Rio Vilas VJ, Birch CP, Johnson J, Kiss IZ, McCarthy ND, Kao RR (2007). Demographic risk factors for classical and atypical scrapie in Great Britain. J Gen Virol.

[B5] Benestad SL, Arsac J-N, Goldmann W, Nöremark M (2008). Atypical/Nor98 scrapie: properties of the agent, genetics, and epidemiology. Vet Res.

[B6] Hoinville LJ, Hoek A, Gravenor MB, McLean AR (2000). Descriptive epidemiology of scrapie in Great Britain: results of a postal survey. Vet Rec.

[B7] Sivam SK, Baylis M, Gravenor MB, Gubbins S, Wilesmith JW (2003). Results of a postal survey in 2002 into the occurrence of scrapie in Great Britain. Vet Rec.

[B8] McIntyre KM, Gubbins S, Sivam SK, Baylis M (2006). Flock-level risk factors for scrapie in Great Britain: analysis of a 2002 anonymous postal survey. BMC Vet Res.

[B9] Tongue SC, Pfeiffer DU, Wilesmith JW (2006). Descriptive spatial analysis of scrapie-affected flocks in Great Britain between January 1993 and December 2002. Vet Rec.

[B10] Dawson M, Vilas VJDR (2008). Control of classical scrapie in Great Britain. In Practice.

[B11] Hopp P, Ulvund MJ, Jarp J (2001). A case-control study on scrapie in Norwegian sheep flocks. Prev Vet Med.

[B12] Healy AM, Hannon D, Morgan KL, Weavers E, Collins JD, Doherty ML (2004). A paired case-control study of risk factors for scrapie in Irish sheep flocks. Prev Vet Med.

[B13] Philippe S, Ducrot C, Roy P, Remontet L, Jarrige N, Calavas D (2005). Sheep feed and scrapie, France. Emerg Infect Dis.

[B14] Gudmundsdottir KB, Sigurdarson S, Kristinsson J, Eiriksson T, Johannesson T (2006). Iron and iron/manganese ratio in forage from Icelandic sheep farms: relation to scrapie. Acta Vet Scand.

[B15] Brown DR (2002). Copper and prion disease. Biochem Soc Trans.

[B16] Chihota CM, Gravenor MB, Baylis M (2004). Investigation of trace elements in soil as risk factors in the epidemiology of scrapie. Vet Rec.

[B17] Imrie CE, Korre A, Munoz-Melendez G (2009). Spatial correlation between the prevalence of transmissible spongiform diseases and British soil geochemistry. Environ Geochem Health.

[B18] Kuchler F, Hamm S (2000). Animal disease incidence and indemnity eradication programs. Agricultural Economics.

[B19] Bowman AW, Azallini A (1997). Applied Smoothing Techniques for Data Analysis: The Kernel Approach with S-Plus Illustrations.

[B20] Scott DW (1992). Multivariate Density Estimation: Theory, Practice, and Visualization.

[B21] Kulldorff M, Nagarwalla N (1995). Spatial disease clusters: detection and inference. Stat Med.

[B22] Diggle PJ, Chetwynd AG (1991). Second-order analysis of spatial clustering for inhomogeneous populations. Biometrics.

[B23] Rowlingson B, Diggle P (1993). Splancs: spatial point pattern analysis code in S-Plus. Computers and Geosciences.

[B24] Breslow NE, Clayton DG (1993). Approximate inference in generalized linear mixed models. Journal of the American Statistical Association.

[B25] Ribeiro PJ, Christensen OF, Diggle PJ (2003). geoR and geoRglm: Software for model-based geostatistics. Proceedings of the 3rd international workshop on distributed statistical computing: 20-22 March 2003 2003; Vienna, Austria.

[B26] Matthews L, Woolhouse ME, Hunter N (1999). The basic reproduction number for scrapie. Proc Biol Sci.

[B27] del Rio Vilas VJ, Guitian J, Pfeiffer DU, Wilesmith JW (2006). Analysis of data from the passive surveillance of scrapie in Great Britain between 1993 and 2002. Vet Rec.

[B28] Jóhannesson T, Gudmundsdóttir KB, Eiríksson T, Kristinsson J, Sigurdarson S (2004). Selenium and GPX activity in blood samples from pregnant and non-pregnant ewes and selenium in hay on scrapie-free, scrapie-prone ad scrapie-afflicted farms in Iceland. Icelandic Agricultural Science.

[B29] Ragnarsdottir KV, Hawkins DP (2006). Bioavailable copper and manganese in soils from Iceland and their relationship with scrapie occurrence in sheep. Journal of Geochemical Exploration.

[B30] Purdey M (2000). Ecosystems suggesting clusters of sporadic TSEs demonstrate excesses of the radical-generating divalent cation manganese and deficiencies of antioxidant cofactors Cu, Se, Fe, Zn. Does a foreign cation substitution at prion protein's Cu domain initiate TSE?. Medical Hypotheses.

[B31] Johnson CJ, Phillips KE, Schramm PT, McKenzie D, Aiken JM, Pedersen JA (2006). Prions adhere to soil minerals and remain infectious. PLoS Pathog.

[B32] Pfeiffer DU, Robinson TP, Stevenson M, Stevens KB, Rogers DJ, Clements ACA (2008). Identifying factors associated with the spatial distribution of disease. Spatial Analysis in Epidemiology.

[B33] Ortiz-Pelaez A, Del Rio Vilas VJ (2009). Within-holding prevalence of sheep classical scrapie in Great Britain. BMC Vet Res.

[B34] Durr PA, Froggatt AEA (2002). How best to geo-reference farms?: A case study from Cornwall, England. Preventive Veterinary Medicine.

[B35] Bailey L, Vardulaki K, Langham J, Chandramohan D (2005). Interpretation of the results of epidemiological studies. Introduction to epidemiology.

